# The Anti-inflammatory Immune Regulation Induced by Butyrate Is Impaired in Inflamed Intestinal Mucosa from Patients with Ulcerative Colitis

**DOI:** 10.1007/s10753-019-01133-8

**Published:** 2019-12-03

**Authors:** Maria K. Magnusson, Stefan Isaksson, Lena Öhman

**Affiliations:** Department of Microbiology and Immunology, Institute of Biomedicine, University of Gothenburg, Box 435, 405 30 Gothenburg, Sweden

**Keywords:** ulcerative colitis, butyrate, mucosal inflammation, immunity

## Abstract

Altered gut microbiota composition and reduced levels of short-chain fatty acids, such as butyrate, have been identified as key components of ulcerative colitis (UC). We aimed to determine and compare effects of butyrate on the intestinal immune profile of UC patients with active disease and non-inflamed controls. Biopsies were cultivated during 6 h with or without butyrate. Cytokines were measured in supernatants and mRNA gene expression was analyzed in biopsies using Qiagen RT^2^ Profiler PCR Arrays. The intestinal immune profile of cultured biopsies, as determined by mRNA gene expression and secreted cytokines, differed between inflamed UC samples and controls. Principal component analysis revealed that addition of butyrate differently regulated mRNA expression in inflamed biopsies from UC and non-inflamed biopsies from controls. Highly discriminant and predictive orthogonal partial least squares discriminant analyses identified 29 genes for UC (R2 = 0.94, Q2 = 0.86) and 23 genes for controls (R2 = 0.90, Q2 = 0.71) that were most regulated by butyrate. UC displayed more up-regulation of genes as compared with controls, and controls displayed the most prominent down-regulations. Ingenuity Pathway Analysis identified a down regulation of the *Neuroinflammation Signaling* pathway and predicted inhibition of the categories *Inflammatory response*, *cellular movement*, and *cellular development* as top diseases and functions, respectively, for controls but not for UC. In conclusion, butyrate has a different effect on gene regulation and more potently down-regulates gene expression of inflammatory pathways in non-inflamed controls than in inflamed tissue of UC patients. These discrepancies may at least partly explain why anticipated anti-inflammatory effects of local butyrate induction or supplementation are not always obtained.

**Electronic supplementary material:**

The online version of this article (10.1007/s10753-019-01133-8) contains supplementary material, which is available to authorized users.

## INTRODUCTION

Inflammatory bowel disease (IBD) is a chronic disease of the gut thought to involve an altered immune response to unknown microbial related threats of genetically susceptible individuals. The composition of the gut microbiota in IBD patients differs from that of healthy individuals, with reports of lower abundance of butyrate-producing bacteria of the Firmicutes phylum such as Roseburia hominis and Faecalibacterium prausnitzii [1, 2]. Further, reduced colonic levels of butyrate, possibly related to disease activity, have been demonstrated in IBD patients [3, 4].

Butyrate, a short-chain fatty acid and the main end-product of intestinal microbial fermentation of dietary fiber in the colon, is a histone deacetylase inhibitor [5] and has been suggested to have anti-inflammatory effects, as shown by the ability to ameliorate disease in animal models of colitis [6–8]. Hence, results primarily obtained from mouse models and *in vitro* systems suggest that butyrate improve intestinal barrier integrity[9], increase secretion of antimicrobial peptides[10, 11], down-regulate Toll-like receptor (TLR) expression and secretion of pro-inflammatory cytokines[12], and inhibit the activity of granulocytes[8] and lymphocytes[13]. Moreover, butyrate may promote T regulatory cell generation [14, 15] and inhibit inflammatory signaling including the AKT [16] and NF-κB pathways[17].

Given its potential anti-inflammatory properties, butyrate has been proposed as a therapeutic option for IBD patients, although results so far have been indecisive. Several clinical trials investigating efficacy of butyrate supplementation suggested the compound to be beneficial for IBD patients[18–21], whereas other studies were not able to show any effect on intestinal inflammation [22, 23]. More recently, attempts to restore the gut microbiota balance by fecal microbiota transplantation (FMT), which may result in increased abundance of butyrate-producing bacterial taxa, have been implemented. A meta-analysis based on randomized controlled trials for patients with UC, including 4 studies with 277 participants, demonstrated that significantly more patients receiving donor FMT achieved clinical remission (42.1%) compared with those receiving control interventions (22.6%), and therefore concluded that FMT appears promising as treatment to induce remission, although long-term durability still remains unclear[24]. Recently, a randomized controlled trial using anaerobically prepared pooled donor FMT or autologous FMT in UC patients reached the primary outcome with a higher likelihood of remission at week 8 in donor compared with autologous FMT [25].

The somewhat diverging results of butyrate induction or supplementation may result from design of studies, and could also be due to that butyrate has different effects on the immune system of the host during inflamed and non-inflamed conditions. Hence, we aimed to determine and compare effects of butyrate on the intestinal immune profile of UC patients with active disease and non-inflamed controls, to potentially identify factors explaining the lack of convincing therapeutic effects on intestinal inflammation of butyrate.

## MATERIALS AND METHODS

### Study Subjects and Sample Collection

Study subjects were recruited among patients at the outpatient clinic at the Sahlgrenska University Hospital, Gothenburg, Sweden. Inflamed (sigmoid colon) and non-inflamed (ascending colon) biopsies were obtained from the same UC patients. Non-inflamed biopsies (sigmoid and ascending colon) were obtained from the same control subjects undergoing colonoscopy for other indications (polyps, weight loss). All biopsies were taken according to a clinical routine using the same diameter endoscopic forceps. Disease activity was determined by Mayo score [26]. The study was performed after receiving informed written consent from all subjects, and the protocol was approved by the Regional Ethical Review Board at the University of Gothenburg.

### Cell Cultivation Assay

Six biopsies were obtained from each site and the four most even-sized were used for cultivation. The same laboratory technician performed the handling of all samples to ensure conformity and all samples were cultivated in duplicates. Biopsies were cultivated in 0.2 mL Iscove’s medium supplemented with 10% human FBS, 100 μg/ml gentamicin (Sigma-Aldrich, St Louis, USA), and 3 μg/ml L-glutamine (Sigma-Aldrich) without or with 1.6 mM butyrate (B5887, Sigma Aldrich), at 37 °C and 5% CO_2_. After 6 h, the duplicate biopsies were collected and pooled for RNA purification and supernatants were pooled and frozen for cytokine analysis. Butyrate concentration was chosen by titration from 0.1 to 6.4 mM on biopsies from healthy controls with reduction of IL-6 expression in the supernatant as readout, lowest concentration exhibiting IL-6 suppression was selected.

### Cytokine Analysis

Quantities of cytokines IL-1β, IL-2, IL-4, IL-5, IL-6, IL-8, IL-10, IL-12p70, IL-13, IL-17A, GM-CSF, TNF, and IFN-γ secreted in culture supernatants were measured by MSD® Multi-Spot Assay system (Meso scale diagnostics, MD, USA), according to the manufacturer’s instructions. Sigmoidal samples were analyzed for all 13 cytokines while samples from ascending colon were analyzed for 8 out of 13 cytokines (IL-1β, IL-2, IL-4, IL-6, IL-8, IL-10, TNF, and IFN-γ).

### Messenger RNA Extraction from Biopsies

Total mRNA from biopsies was extracted using Nucleospin® DNA, RNA, and protein purification kit (Macherey-Nagel, Düren, Germany) according to the manufacturer’s protocol. Concentration and purity of the RNA was measured using a NanoDrop ND-1000 spectrophotometer (NanoDrop Technologies, Wilmington, Delaware, USA) with 260/280 and 260/230 ratios of ~ 2 and 2.1–2.2, respectively.

### Mucosal Gene Expression Array Analysis

Gene expression array analysis was performed as previously described [27]. Briefly, cDNA was prepared using the RT^2^ First Strand Kit (Qiagen, Hilden, Germany) according to the manufacturer’s protocol. The RT^2^ Profiler PCR arrays for “antibacterial response” (PHAS-148Z, Qiagen) and “innate and adaptive immune responses” (PAHS-052Z, Qiagen) were analyzed in a Quantstudio 12K Flex Real-Time PCR System (Applied Biosystems, Foster City, CA, USA) by the use of RT^2^ qPCR SYBR Green ROX MasterMix (Qiagen). Data was analyzed in the RT^2^ Profiler PCR Array Data Analysis version 3.5 (Qiagen). All samples passed the quality checks for PCR array reproducibility, RT efficiency, and genomic DNA contamination. ACTB, B2M, and RPLP0 were used as housekeeping genes and normalized values are expressed as 2^−(Target-Housekeeping)^. For genes present in both arrays (*n* = 34), mean values from the two arrays were used. A complete list of the genes from the arrays, including annotation of duplicates and undetected genes (*n* = 14), is shown in Supplementary table 1.

### Data Analyses

Principle component analysis (PCA) was conducted on logarithm-transformed gene expression data using the prcomp-algorithm and visualized using the pca3d-package in R (version 3.3.2) [28].

To examine the relationship between gene expression without and with butyrate (Y-variable) and mRNA levels (X-variables), multivariate factor analysis (SIMCA-P+ software; Umetrics, Umeå, Sweden) was used. Orthogonal Projections to Latent Structures Discriminant Analyses (OPLS-DA) were implemented to correlate selected *Y*-variables and *X*-variables with each other in linear multivariate models. The quality of the OPLS-DA was based on the parameters R2, that is, the goodness of the fit of the model (values of ≥ 0.5 defines good discrimination, best possible fit, R2 = 1) and Q2, that is, the goodness of prediction of the model (values of ≥ 0.4 defines high predictive ability, best possible predictive ability Q2 = 1).[29] A variable influence on projection (VIP) cut-off of > 1.2 was used as a variable selection based on discriminatory power, since VIP > 1 is most influential for the model and most relevant for explaining the Y observations[29]. Significance of the separation between the groups in the OPLS-DA was calculated using CV-ANOVA.

Parameters identified by OPLS-DA as VIP > 1.2 were logarithm transformed and subject to univariate analysis using Student’s *T* test. Mean values were back transformed and fold changes (FC) were calculated between butyrate 1.6 mM and none for each gene and evaluated using Ingenuity Pathway Analysis (IPA, version 2.3) (Qiagen). Cut-off used for IPA was *p* < 0.05. Gene expression data are presented as FC on a log_2_ scale where log_2_FC = 1 and − 1 define a 2-fold increase and decrease, respectively. Activation *z*-scores are calculated by the IPA software and predict whether a specific pathway, disease or function is increased (*z*-score > 2.0) or decreased (*z*-score < − 2.0) based on the experimental dataset.

For cytokine expression, the Mann-Whitney *U* test was used to determine differences between two independent groups and Wilcoxon signed-rank test was used for comparison of related samples. Data are shown as median (range) unless otherwise stated.

All statistical analyses were performed using IBM SPSS Statistics 23; *p* values < 0.05 were considered as statistically significant. Boxplots and the heatmap were generated using the ggplot2-package in R.

## RESULTS

### Patient Demographics

The study included 8 controls (4 males, age 54 (24–84), median (range)) and 8 patients with UC (6 males, age 38 (32–74)) with a disease duration of UC of 7 (< 1–47) years. All UC patients had active disease with sigmoidal endoscopic Mayo score 2 (*n* = 7) or 1 (*n* = 1) and Mayo score 0 in ascending colon. All controls had endoscopic Mayo score 0 throughout the colon. Current treatment for UC patients was 5ASA (*n* = 5), 5ASA and thiopurines (*n* = 1), corticosteroids (*n* = 1), and no treatment (*n* = 1). None of the controls was taking any anti-inflammatory drugs and none of the participants had used antibiotics the past 3 months.

### UC Patients Show Increased Biopsy Cytokine Secretion and Innate/Adaptive Gene Expression at the Site of Inflammation

**Fig. 1:**
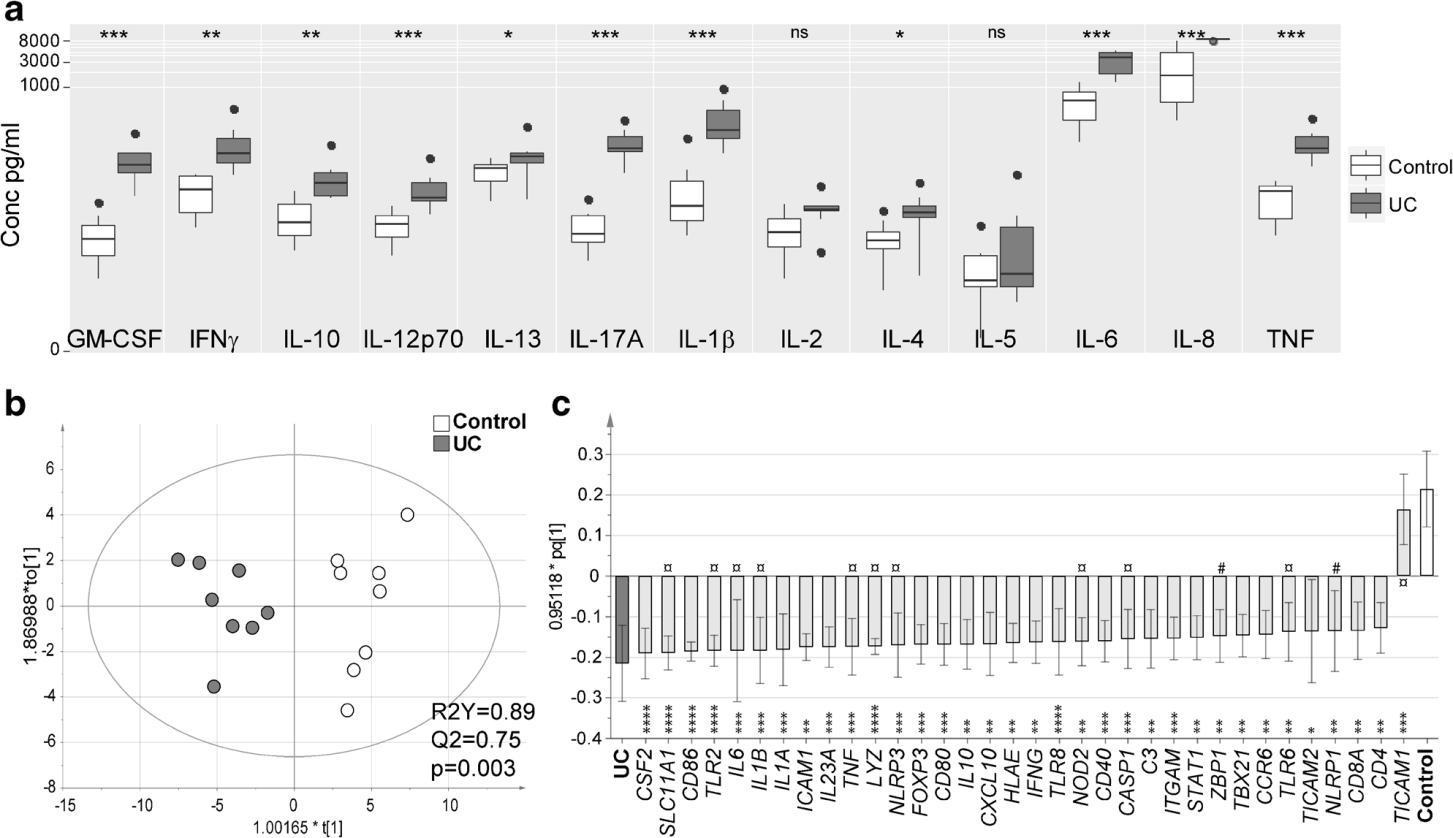
Cytokine protein expression and gene expression from *in vitro* cultivated biopsies. Sigmoidal biopsies were taken from control subjects (*n* = 8, shown in white) and UC patients (*n* = 8, shown in gray) and cultivated *in vitro* for 6 h. Cytokine levels in the supernatants were analyzed by MSD® Multi-Spot Assay system and gene expression was analyzed using PCR arrays for genes involved in antibacterial response and innate and adaptive immune responses. **a** Levels of GMCSF, IFN-γ, IL-10, IL-12p70, IL-13, IL-17A, IL-1β, IL-2, IL-4, IL-5, IL6, IL-8, and TNF in the supernatants. **b** Score scatter plot and **c** loading column plot from an Orthogonal Projections to Latent Structures Discriminant Analysis (OPLS-DA) for the expressed genes using a variable influence on projection (VIP) cut-off of > 1.2. Genes in **c** are shown in light gray, genes present in both arrays (“antibacterial response” and “innate and adaptive immune responses”) are marked with ¤, genes present exclusively in “antibacterial response” are marked with #, and genes present exclusively in “innate and adaptive immune responses” are unmarked. Significance was assessed by Mann-Whitney *U* test (cytokine data) or Student’s *T* test (logarithm-transformed gene data); *p < 0.05, **p < 0.01, ***p < 0.001, and ****p < 0.0001.

Cytokine analysis of the sigmoidal biopsy supernatants showed increased secretion of all cytokines except IL-2 and IL-5 for UC patients as compared with controls (Fig. 1a), and a similar pattern was apparent when compared with non-inflamed biopsies from the same UC patients (Supplementary Figure 1). The mRNA expression in sigmoidal biopsies cultured without butyrate revealed higher expression of genes mainly present in the array “innate and adaptive immune response” for UC patients compared with controls, with only *NLRP1* and *ZBP1* related exclusively to the “antibacterial response” array (Fig. 1b, c).

### Butyrate Modifies Cytokine Secretion Differently at the Site of Inflammation Compared with Non-inflamed Sites

**Fig. 2:**
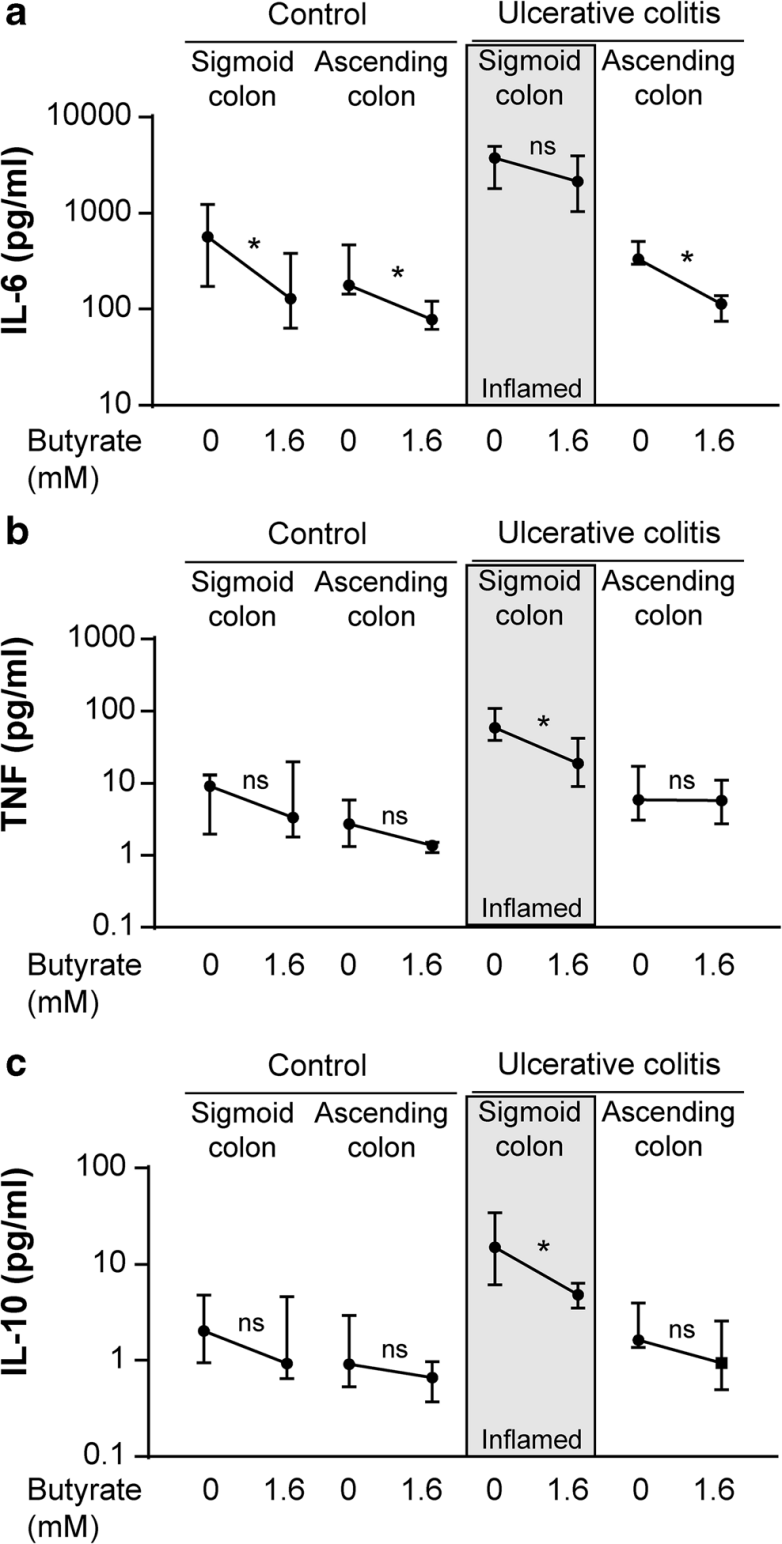
Alterations in cytokine secretion induced by butyrate in colonic biopsies. Biopsies were taken from sigmoid and ascending colon from control subjects (*n* = 8) and UC patients (*n* = 8) and cultivated *in vitro* for 6 h without or with 1.6 mM butyrate. Cytokine levels in the supernatants were analyzed by MSD® Multi-Spot Assay system. Levels of IL-6 (**a**), TNF (**b**), and IL-10 (**c**) are shown as median and interquartile range and samples from inflamed tissue are highlighted with a gray background. Significance was assessed by Wilcoxon signed-rank test; *p < 0.05.

Addition of butyrate for 6 h decreased the levels of IL-6 for controls and non-inflamed samples from UC patients but no changes were detected for inflamed UC samples (Fig. 2a). The opposite pattern was identified for TNF and IL-10 where butyrate only induced a decrease for inflamed UC samples and not at non-inflamed sites or for controls (Fig. 2b, c). In addition, GM-CSF was reduced by butyrate for controls (0.94 pg/ml (0.15–4.73) to 0.22 pg/ml (0.06–0.42), *p* = 0.02), while no other changes for any of the cytokines could be detected at any site (data not shown).

### Butyrate Alters the Sigmoidal Gene Expression Profile of Both UC Patients and Controls

**Fig. 3:**
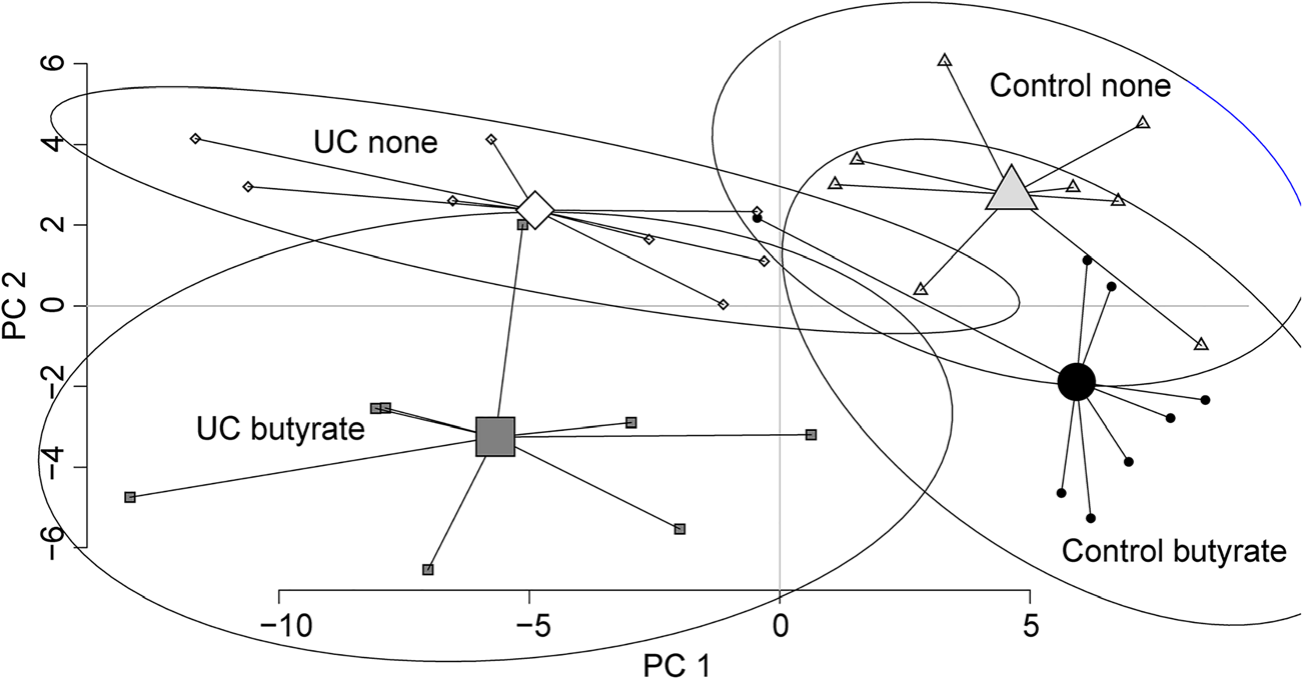
Overview of mucosal gene expression in controls and patients with UC, with and without butyrate. Sigmoidal biopsies were taken from control subjects (*n* = 8) and UC patients (*n* = 8) and cultivated *in vitro* for 6 h without and with 1.6 mM butyrate. Gene expression was analyzed using PCR arrays for genes involved in antibacterial response and innate and adaptive immune responses. The data is presented by a principle component analysis including all detectable genes (*n* = 120). Controls without butyrate (control none, gray triangles), controls with butyrate (control butyrate, black circles), UC without butyrate (UC none, white diamonds), and UC with butyrate (UC butyrate, gray squares) are shown. Groups are connected by lines to their centroids and the confidence intervals (0.95%) are shown by the ellipses.

**Fig. 4:**
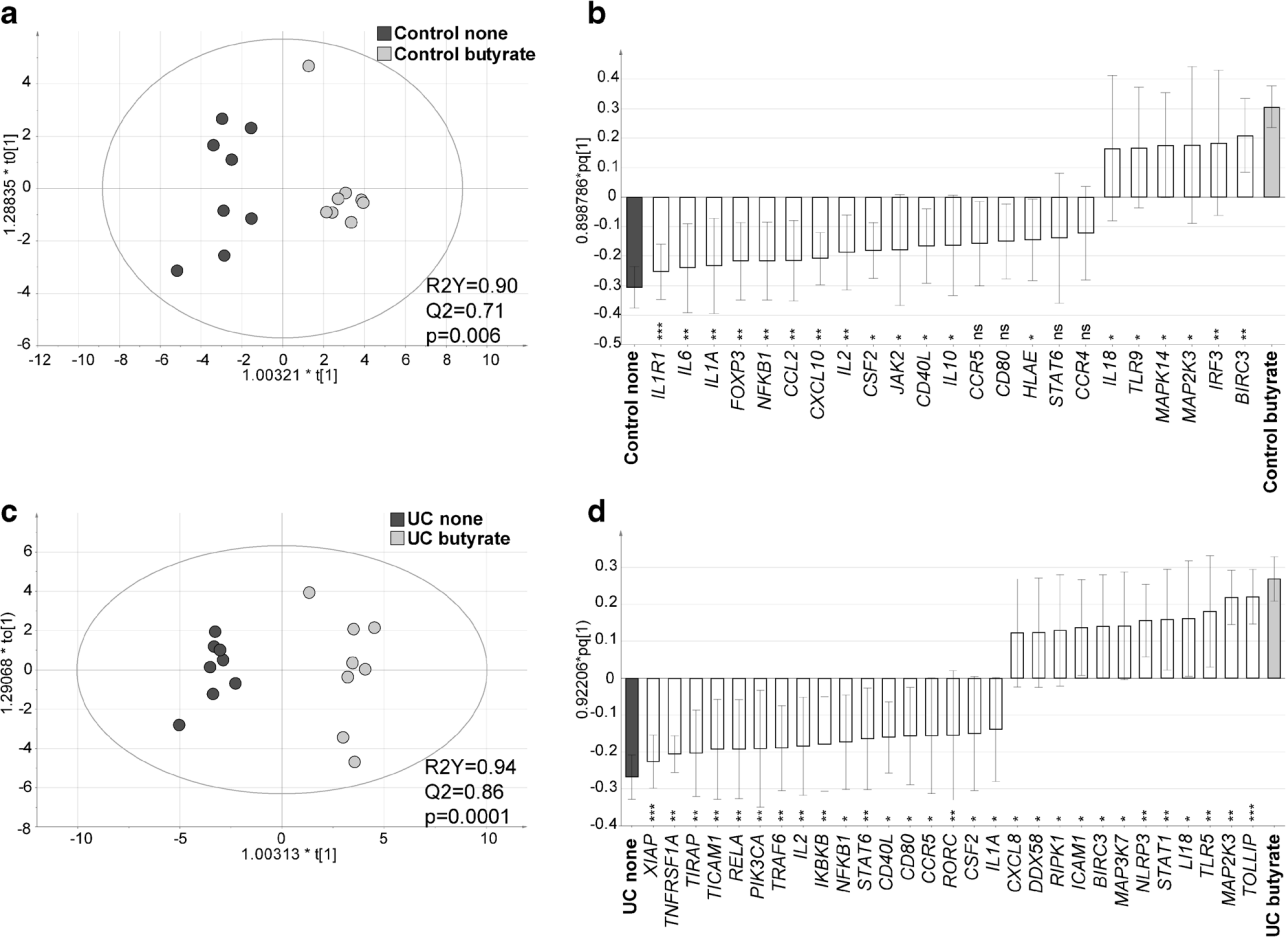
Butyrate induces differential mucosal regulation of gene expression in controls and patients with UC. Sigmoidal biopsies were taken from control subjects (*n* = 8) and UC patients (*n* = 8) and cultivated *in vitro* for 6 h without and with 1.6 mM butyrate. Gene expression was analyzed using PCR arrays for genes involved in antibacterial response and innate and adaptive immune responses. Orthogonal Projections to Latent Structures Discriminant Analysis (OPLS-DA) with gene expression as *X*-variables (shown in white) and patient groups as *Y*-variables (without butyrate shown in black and with butyrate shown in gray) are depicted. **a** Score scatter plot and **b** loading scatter plot from an OPLS-DA for controls. **c** Score scatter plot and **d** loading scatter plot from an OPLS-DA for patients with UC. Variable influence on projection (VIP) cutoffs of 1.2 were used. R2Y defines the goodness of fit and Q2 the goodness of prediction. Significance of regulated genes in **c** and **d** was assessed by Student’s *T* test, *p < 0.05, **p < 0.01, ***p < 0.001 and ****p < 0.0001. ns, not significant.

The mRNA expression of inflamed sigmoidal biopsies from UC patients and non-inflamed sigmoidal biopsies from controls cultured with or without butyrate revealed differential clustering in a PCA, reflecting different regulation of gene expression by the addition of butyrate at inflammation (Fig. 3). Highly discriminant and predictive OPLS-DA plots identified 23 genes for controls (R2 = 0.90, Q2 = 0.71, *p* = 0.006) and 29 genes for UC (R2 = 0.94, Q2 = 0.86, *p* = 0.0001) that were most regulated by butyrate (Fig. 4). Of the genes identified to be regulated by butyrate in the OPLS-DA analysis, 11 were overlapping and one (*CCR4*) showed no significant change within any of the groups resulting in a combination of 40 genes used for further analysis (Fig. 4b, d).

### Ingenuity Pathway Analysis Indicates More Potent Down-regulation of Inflammatory Pathways for Controls than for UC Patients at the Site of Inflammation

**Fig. 5:**
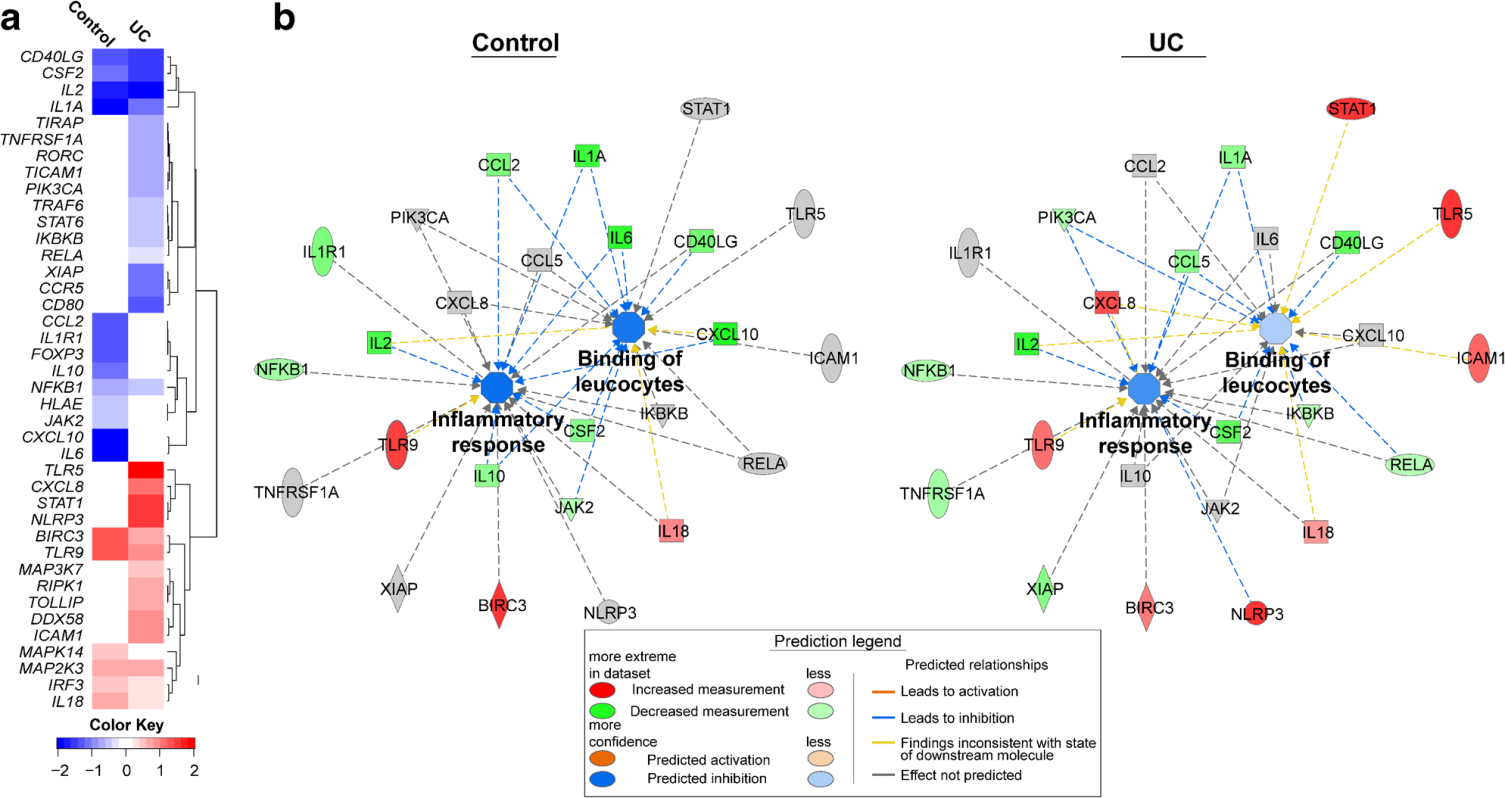
Evaluation of genes regulated by butyrate in controls and patients with UC. Gene expression was analyzed using PCR arrays for genes involved in antibacterial response and innate and adaptive immune responses in sigmoidal biopsies; data was logarithm transformed, mean values for significantly regulated genes were back transformed, and Log2 fold changes (FC) were calculated between butyrate 1.6 mM and none for each gene in controls (*n* = 8) and patients with UC (*n* = 8). **a** Heatmap displaying changes in biopsy gene expression by 1.6 mM butyrate. FC are displayed as colors ranging from blue (down-regulation) to red (up-regulation) as shown in the key and columns are clustered using correlation distance. Non-significant regulations are shown in white. **b** Visual representation of the analyzed genes in prediction of the biological functions *Inflammatory response* and *Binding of leucocytes* using Ingenuity Pathway Analysis (IPA). Analyses were performed for controls (left) and patients with UC (right). Up-regulated genes are shown in red, down-regulated genes in green, and detected but not significantly regulated genes in gray. The intensity of the color in a node indicates the degree of up- or down-regulation. Orange lines and nodes indicate predicted up-regulation, whereas blue lines and nodes indicate predicted down-regulation. The intensity of the color in a node indicates the degree of predicted up- or down-regulation. Broken edge lines indicate indirect relationship. The shapes of the nodes reflect the functional class of each gene product: transcriptional regulator (horizontal ellipse), transmembrane receptor (vertical ellipse), enzyme (rhombus), cytokine/growth factor (square), kinase (triangle), and complex/group/other (circle).

Fold changes in gene expression caused by butyrate in sigmoidal biopsies were analyzed in a heatmap. Common for both groups were down-regulation of *IL2*, *IL1A*, *CSF2*, *CD40LG*, and *NFKB1* and up-regulation of *BIRC3*, *TLR9*, *MAP2K3*, *IRF3*, and *IL18*, while regulation of the other genes differed (Fig. 5a). Overall, UC displayed more up-regulation of genes as compared with controls, as shown by genes indicated in red, and controls displayed the most prominent down-regulations, as shown by genes indicated in blue (Fig. 5a). Next, the regulated genes were evaluated by IPA. The top canonical pathway identified by IPA was *Neuroinflammation Signaling* pathway (-log(*p* value) 39, 27 associated genes), and *z*-scores of − 2.117 and − 0.962 for controls and UC patients, respectively, indicated a down-regulation in the presence of butyrate for controls but not for UC patients. Data were further analyzed with the IPA software to identify the most significantly enriched diseases and functions related to gene regulation by butyrate. Among the top eight most enriched diseases and functions for controls and UC, controls showed a decrease in predicted activation states for 5 diseases/functions (defined by a *z*-score < − 2.0) while no changes were detected for patients with UC (Table 1). Since inflammatory response and leucocyte movement/interaction were enriched for both groups, the changes induced by butyrate were further visualized in relation to the biological functions of *Inflammatory response* and *Binding of leucocytes*. IPA predicted strong inhibition of both functions for controls as shown by the dark blue color of the nodes connecting the analyzed genes (Fig. 5b, left). For UC patients at inflammation, only modest and weak inhibition were predicted for *Inflammatory response* and *Binding of leucocytes*, respectively, as shown by the medium and light blue colors of the connecting nodes (Fig. 5b, right).

**Table 1:** Top Enriched Diseases and Functions Determined Using Ingenuity Pathway Analysis (IPA). Genes Regulated by Butyrate Were Analyzed with IPA Software to Outline the Most Enriched Diseases and Functions. Top eight Enriched Terms for Controls and Ulcerative Colitis (UC) Are Shown Along with the Overlapping Number of Genes in the Datasets, *p* values, and Activation *z*-scores

Category	Diseases/functions	#genes	*p* value	*z*-score^a^
Controls				
Inflammatory response	Inflammatory response	14	3.69E−20	**−***2.111*^b^
Cellular movement	Cell movement of leukocytes	9	1.26E−17	**−***2.429*
Cell death and survival	Apoptosis of leukocytes	11	1.10E−16	0.435
Cell-to-cell signaling and interaction	Adhesion of immune cells	10	1.58E−15	− 1538
Cell death and survival	Apoptosis of phagocytes	8	5.15E−15	0.432
Cell-to-cell signaling and interaction	Stimulation of cells	11	1.25E−14	**−***2.318*
Cell-to-cell signaling and interaction	Stimulation of blood cells	7	5.72E−14	**−***2.038*
Connective tissue development and function	Growth of connective tissue	10	7.77E−14	**−***2.284*
Patients with UC				
Cell death and survival	Apoptosis	27	5.71E−21	1.417
Cell-to-cell signaling and interaction	Interaction of leukocytes	14	3.06E−19	− 0.616
Inflammatory response	Inflammatory response	16	3.36E−19	− 1.127
Cell death and survival	Cell death of tumor cell lines	24	1.19E−17	1.146
Cell-to-cell signaling and interaction	Binding of leukocytes	13	1.80E−17	− 0.419
Cell death and survival	Cell death of Ag presenting cells	9	1.97E−16	1.719
Cell-to-cell signaling and interaction	Adhesion of immune cells	12	2.35E−16	− 0.031
Gene expression	Transactivation	15	4.58E−16	− 1.132

^a^Activation *z*-score is calculated by the IPA software and predicts whether a specific disease or bio-function is increased (positive *z*-score) or decreased (negative *z*-score) based on the experimental dataset. *Z*-scores < − 2 or > 2 indicates decrease and increase, respectively

^b^*Z*-scores of < − 2 or > 2 are shown in italic

IPA, Ingenuity Pathway Analysis; UC, ulcerative colitis

## DISCUSSION

In this study, we have demonstrated that butyrate alters the intestinal gene expression profile of both UC patients and non-inflamed controls. Nevertheless, butyrate had a different effect on intestinal gene expression during inflamed and non-inflamed conditions. Hence, butyrate more potently down-regulated expression of inflammatory pathways in intestinal biopsies from non-inflamed controls than in inflamed biopsies from UC patients.

In the absence of butyrate, the cytokine secretion and gene expression of cultured sigmoidal biopsies differed substantially between the two study groups. The higher cytokine secretion as well as expression of genes encoding pro-inflammatory cytokines, chemokines, and receptors for recognition of microbial products in inflamed UC patients than non-inflamed controls reflected the inflammatory status of UC patients with active disease. Few changes were detected in cytokine expression due to butyrate, which was not surprising given the short incubation time. However, paired samples from inflamed and non-inflamed tissue of the UC patients as well as paired samples from sigmoid and ascending colon from controls revealed that the few butyrate-induced differences in cytokine secretion were inherent to the inflammation, not to the disease. Undeniably, butyrate had similar effects on the regulation of the expression of a smaller share of genes in UC patients and non-inflammatory controls. Similarities of gene regulation in the presence of butyrate in the two groups included down-regulated expression of *NFKB1*, along with reduced secretion of the NF-κB-induced pro-inflammatory cytokines TNFα and IL6. Indeed, in CD patients, butyrate has been demonstrated to inhibit NF-κB activation and IκBα degradation [30] and control ROS-mediated NF-kB activation [31]. Also, genes expressed by T regulatory cells, i.e., RORC and FOXP3, were down-regulated by butyrate in UC patients and non-inflammatory controls, respectively. While butyrate has been reported to support induction of FOXP3^+^ T regulatory cells in animal models [14, 15], data from the human setting are so far lacking, and our results do not support butyrate-induced expression of genes related to this admittedly anti-inflammatory cell population. Furthermore, the gene expression of a few cytokines and growth factors was regulated similarly in both groups. Hence, the effect of butyrate on the expression of a limited numbers of genes is shared during inflamed and non-inflamed conditions.

The most prominent finding of our study was that butyrate had different effects on regulation of gene expression with only minor overlaps between the non-inflamed controls and UC patients. Based on the intestinal gene expression, IPA identified *Neuroinflammation Signaling* pathway to be inhibited in non-inflamed controls, but not in UC patients with active disease. IPA also predicted different regulation of the biological diseases and functions *Inflammatory response* and *Binding of leucocytes* for the two groups with a stronger decrease for controls*.* Hence, our data suggest that butyrate more prominently down-regulate genes alluring to inflammatory pathways during the healthy state, but not during chronic inflammatory conditions.

There are few previous studies of the individual genes that were differently regulated by the presence of butyrate in health and disease. Nevertheless, XIAP expression, found to be down-regulated by butyrate in UC patients but not in non-inflamed controls, has been reported to be reduced by butyrate addition to Caco-2 cells [32]. Additionally, similar to our finding, butyrate has been reported to substantially down-regulate the expression of CD80 on human dendritic cells [33]. Opposite to reports of butyrate having effects on epithelial function expression of antimicrobial peptides such as cathelecidin [11], RegIIIγ, and β-defensins [10, 34], we found no evidence for regulation of genes encoding for antimicrobial peptides. Potentially, the conflicting results may reflect that previous studies have made use of epithelial cell lines, whereas our data were based on cultured biopsies, where the epithelial barrier only constitutes a smaller part of the whole tissue. Still, the effect of butyrate on the human intestinal immune profile, irrespective of presence of inflammation, is under-investigated and needs further attention.

The altered regulation of genes of several inflammatory pathways in UC patients with active inflammation as described in our study may be due to impaired butyrate metabolism. Possibly, inflammatory conditions can result in down-regulated expression of genes encoding enzymes involved in butyrate metabolism and oxidation, as demonstrated in inflamed mucosa of UC patients [35]. This is supported by the fact that in UC patients responding to anti-TNF therapy the mucosal expression of butyrate metabolism/oxidation genes are increased [35]. Another study, promoting that butyrate has different effects on intestinal cells during health and disease, demonstrated that addition of butyrate alone to intestinal primary epithelial cultures increased the epithelial resistance, whereas butyrate in the presence of pro-inflammatory cytokines led to a drop in resistance [36]. In line with this, a recent study showed that TNFα reduces the ability of the intestinal epithelium to take up and metabolize butyrate [37]. Hence, others and our data imply that butyrate effects on the host are altered by inflammation and butyrate supplementation alone may be insufficient to resolve inflammation and regain gut homeostasis.

Recently, manipulation of the gut microbiota by fecal transplantation, potentially increasing the abundance of butyrate-producing taxa, has been proposed as a target for IBD therapy [38]. So far, the treatment efficacy seems highly variable and may be related to microbiota composition of the donor and/or recipient. Interestingly, sustained remission in UC patients undergoing fecal transplantation has been associated with increased abundance of butyrate-producing bacteria and overall increased butyrate production capacity, while relapse was associated with persisting high relative abundance of Proteobacteria and Bacteroidetes [39]. Our data, along with the reports of impaired butyrate metabolism during inflammatory conditions described above, imply that improved long-term effects of butyrate induction, or local supplementation, may be best achieved while distributing butyrate during non-inflamed conditions. Hence, butyrate may be more potent to maintain rather than induce remission.

The fact that we only determined the effects of butyrate on intestinal gene expression, not including analyses of certain cell populations or protein expression, is a potential weakness of this study. Further, the number of study subjects included is limited and addition of consecutive time-points or several concentrations of butyrate for our analyses might have revealed another pattern of gene regulation and cytokine secretion by the addition of butyrate. Indeed, underdosing may be a reason for the differences detected in our study and in clinical studies of butyrate supplementation. However, our approach, analyzing pathway-focused gene expression using verified PCR assays, allows for a broad and still relatively detailed analysis including a wide range of targets in specific pathways. Also, coherent analysis methods, including multivariate tools and Ingenuity Pathway Analysis, admit for better understanding of the overall picture, rather than isolated findings. To the best of our knowledge, this study is the first of its kind, comparing effects of butyrate on human gene expression *ex vivo*, making use of intestinal biopsy cultures during inflammatory conditions in ulcerative colitis patients and non-inflammatory controls.

To conclude, although butyrate altered the intestinal gene expression profile of both UC patients and non-inflamed controls, butyrate regulated a different set of genes during inflamed and non-inflamed conditions. Inflammatory pathways were more potently down-regulated by butyrate in non-inflamed controls than UC patients at active inflammation. These discrepancies may at least partly explain why anticipated anti-inflammatory effects of local butyrate induction or supplementation in UC patients are not always obtained.

## Electronic Supplementary Material

Supplementary Figure 1. Cytokine protein expression from *in vitro* cultivated biopsies in UC patients. Inflamed sigmoidal biopsies and non-inflamed biopsies from ascending colon were taken from UC patients (n = 8) and cultivated *in vitro* for 6 h. Cytokine levels in the supernatants were analyzed by MSD® Multi-Spot Assay system. Levels of IFN-γ, IL-10, IL-1β, IL-2, IL-4, IL6, IL-8 and TNF in the supernatants are shown from inflamed tissue (grey) and non-inflamed tissue (white). Significance was assessed by Mann Whitney U test; **p < 0.01 and ***p < 0.001. (PNG 61 kb)

High resolution image (TIF 255 kb)

Supplementary Table 1 (DOCX 48 kb)
