# Supplementary figures and images for: The Anti-inflammatory Immune Regulation Induced by Butyrate Is Impaired in Inflamed Intestinal Mucosa from Patients with Ulcerative Colitis
Source: Inflammation. 2019 Dec 3;43(2):507–17. doi: 10.1007/s10753-019-01133-8 (PMC7170981; doi:10.1007/s10753-019-01133-8)

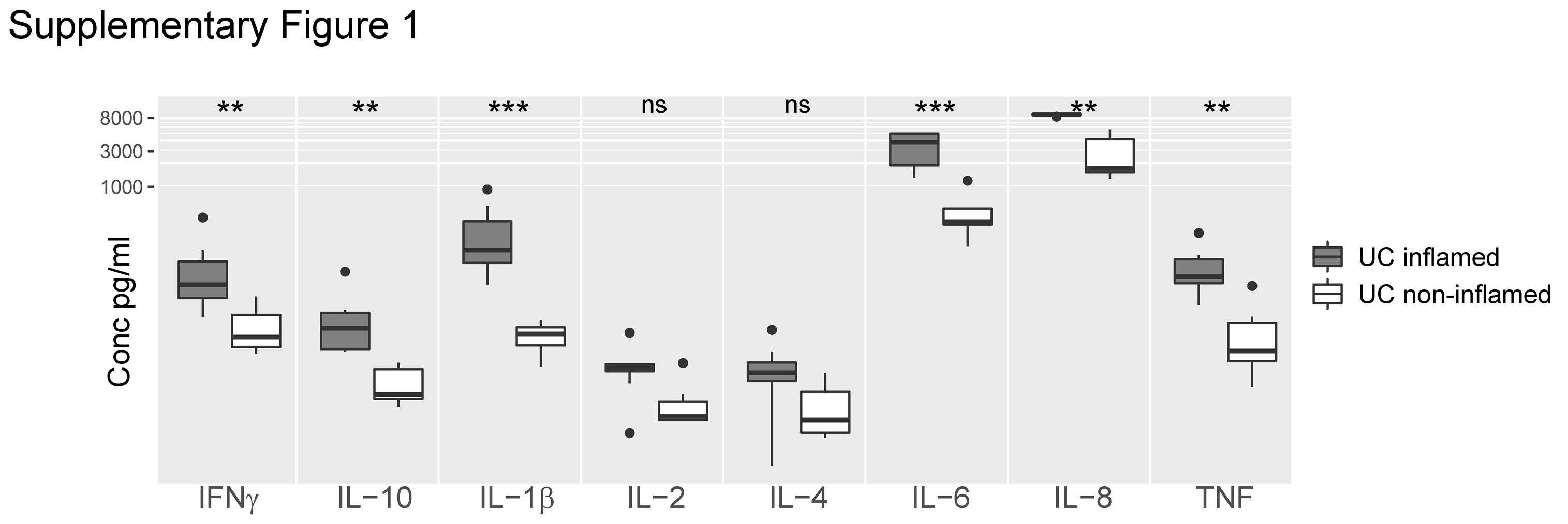

Supplement: Supplementary file 1 — Cytokine protein expression from in vitro cultivated biopsies in UC patients. Inflamed sigmoidal biopsies and non-inflamed biopsies from ascending colon were taken from UC patients (n = 8) and cultivated in vitro for 6 h. Cytokine levels in the supernatants were analyzed by MSD® Multi-Spot Assay system. Levels of IFN-γ, IL-10, IL-1β, IL-2, IL-4, IL6, IL-8 and TNF in the supernatants are shown from inflamed tissue (grey) and non-inflamed tissue (white). Significance was assessed by Mann Whitney U test; **p < 0.01 and ***p < 0.001. (PNG 61 kb) [file 10753_2019_1133_Fig6_ESM.png]

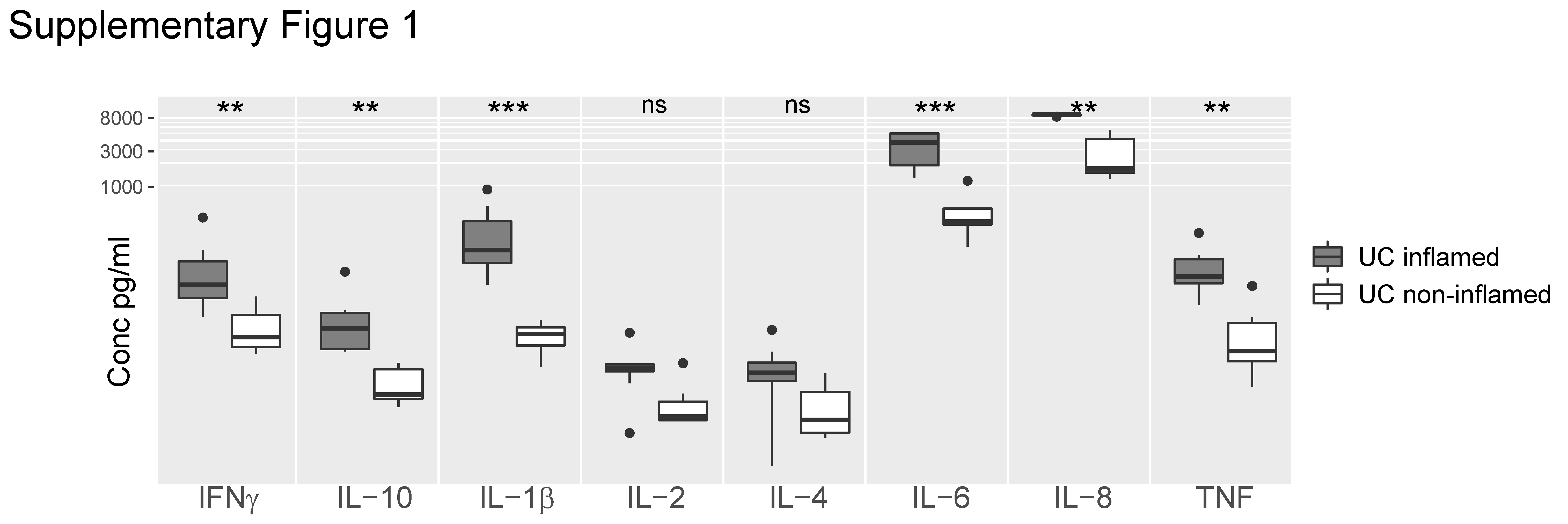

Supplement: Supplementary file 2 — High resolution image (TIF 255 kb) [file 10753_2019_1133_MOESM1_ESM.tif]
