# Supplementary material for: The Anti-inflammatory Immune Regulation Induced by Butyrate Is Impaired in Inflamed Intestinal Mucosa from Patients with Ulcerative Colitis
Source: Inflammation. 2019 Dec 3;43(2):507–17. doi: 10.1007/s10753-019-01133-8 (PMC7170981; doi:10.1007/s10753-019-01133-8)
Supplement: Supplementary file 3 — (DOCX 48 kb) [file 10753_2019_1133_MOESM2_ESM.docx]

**SUPPLEMENTART TABLE 1.** Description of genes included in the RT^2^ Profiler PCR arrays “Antibacterial response” and “Innate and Adaptive Immune Responses”.

|  | | *Antibacterial response array* |  |
| --- | --- | --- | --- |
| **Gene name** | | **Description** |  |
| *AKT1* | | V-akt murine thymoma viral oncogene homolog 1 |  |
| *APCS*^#^* | | Amyloid P component, serum |  |
| *BIRC3* | | Baculoviral IAP repeat containing 3 |  |
| *BPI^#^* | | Bactericidal/permeability-increasing protein |  |
| *CAMP^#^* | | Cathelicidin antimicrobial peptide |  |
| *CARD6* | | Caspase recruitment domain family, member 6 |  |
| *CARD9* | | Caspase recruitment domain family, member 9 |  |
| *CASP1** | | Caspase 1, apoptosis-related cysteine peptidase (interleukin 1, beta, convertase) |  |
| *CASP8* | | Caspase 8, apoptosis-related cysteine peptidase |  |
| *CCL3* | | Chemokine (C-C motif) ligand 3 |  |
| *CCL5** | | Chemokine (C-C motif) ligand 5 |  |
| *CD14** | | CD14 molecule |  |
| *CHUK* | | Conserved helix-loop-helix ubiquitous kinase |  |
| *CRP*^#^* | | C-reactive protein, pentraxin-related |  |
| *CTSG* | | Cathepsin G |  |
| *CXCL1* | | Chemokine (C-X-C motif) ligand 1 (melanoma growth stimulating activity, alpha) |  |
| *CXCL2* | | Chemokine (C-X-C motif) ligand 2 |  |
| *DMBT1* | | Deleted in malignant brain tumors 1 |  |
| *FADD* | | Fas (TNFRSF6)-associated via death domain |  |
| *HSP90AA1* | | Heat shock protein 90kDa alpha (cytosolic), class A member 1 |  |
| *IFNA1*^#^* | | Interferon, alpha 1 |  |
| *IFNB1*^#^* | | Interferon, beta 1, fibroblast |  |
| *IKBKB* | | Inhibitor of kappa light polypeptide gene enhancer in B-cells, kinase beta |  |
| *IL12A* | | Interleukin 12A (natural killer cell stimulatory factor 1, cytotoxic lymphocyte maturation factor 1, p35) |  |
| *IL12B^#^* | | Interleukin 12B (natural killer cell stimulatory factor 2, cytotoxic lymphocyte maturation factor 2, p40) |  |
| *IL18** | | Interleukin 18 (interferon-gamma-inducing factor) |  |
| *IL1B** | | Interleukin 1, beta |  |
| *IL6** | | Interleukin 6 (interferon, beta 2) |  |
| *CXCL8** | | Interleukin 8 |  |
| *IRAK1** | | Interleukin-1 receptor-associated kinase 1 |  |
| *IRAK3* | | Interleukin-1 receptor-associated kinase 3 |  |
| *IRF5* | | Interferon regulatory factor 5 |  |
| *IRF7** | | Interferon regulatory factor 7 |  |
| *JUN* | | Jun proto-oncogene |  |
| *LBP^#^* | | Lipopolysaccharide binding protein |  |
| *LCN2* | | Lipocalin 2 |  |
| *LTF* | | Lactotransferrin |  |
| *LY96** | | Lymphocyte antigen 96 |  |
| *LYZ** | | Lysozyme |  |
| *MAP2K1* | | Mitogen-activated protein kinase kinase 1 |  |
| *MAP2K3* | | Mitogen-activated protein kinase kinase 3 |  |
| *MAP2K4* | | Mitogen-activated protein kinase kinase 4 |  |
| *MAP3K7* | | Mitogen-activated protein kinase kinase kinase 7 |  |
| *MAPK1** | | Mitogen-activated protein kinase 1 |  |
| *MAPK14* | | Mitogen-activated protein kinase 14 |  |
| *MAPK3* | | Mitogen-activated protein kinase 3 |  |
| *MAPK8** | | Mitogen-activated protein kinase 8 |  |
| *MEFV* | | Mediterranean fever |  |
| *MPO*^#^* | | Myeloperoxidase |  |
| *MYD88** | | Myeloid differentiation primary response gene (88) |  |
| *NAIP* | | NLR family, apoptosis inhibitory protein |  |
| *NFKB1** | | Nuclear factor of kappa light polypeptide gene enhancer in B-cells 1 |  |
| *NFKBIA** | | Nuclear factor of kappa light polypeptide gene enhancer in B-cells inhibitor, alpha |  |
| *NLRC4* | | NLR family, CARD domain containing 4 |  |
| *NLRP1* | | NLR family, pyrin domain containing 1 |  |
| *NLRP3** | | NLR family, pyrin domain containing 3 |  |
| *NOD1** | | Nucleotide-binding oligomerization domain containing 1 |  |
| *NOD2** | | Nucleotide-binding oligomerization domain containing 2 |  |
| *PIK3CA* | | Phosphoinositide-3-kinase, catalytic, alpha polypeptide |  |
| *PRTN3^#^* | | Proteinase 3 |  |
| *PSTPIP1* | | Proline-serine-threonine phosphatase interacting protein 1 |  |
| *PYCARD* | | PYD and CARD domain containing |  |
| *RAC1* | | Ras-related C3 botulinum toxin substrate 1 (rho family, small GTP binding protein Rac1) |  |
| *RELA* | | V-rel reticuloendotheliosis viral oncogene homolog A (avian) |  |
| *RIPK1* | | Receptor (TNFRSF)-interacting serine-threonine kinase 1 |  |
| *RIPK2* | | Receptor-interacting serine-threonine kinase 2 |  |
| *SLC11A1** | | Solute carrier family 11 (proton-coupled divalent metal ion transporters), member 1 |  |
| *SLPI* | | Secretory leukocyte peptidase inhibitor |  |
| *SUGT1* | | SGT1, suppressor of G2 allele of SKP1 (S. cerevisiae) |  |
| *TICAM1** | | Toll-like receptor adaptor molecule 1 |  |
| *TICAM2* | | Toll-like receptor adaptor molecule 2 |  |
| *TIRAP* | | Toll-interleukin 1 receptor (TIR) domain containing adaptor protein |  |
| *TLR1** | | Toll-like receptor 1 |  |
| *TLR2** | | Toll-like receptor 2 |  |
| *TLR4** | | Toll-like receptor 4 |  |
| *TLR5** | | Toll-like receptor 5 |  |
| *TLR6** | | Toll-like receptor 6 |  |
| *TLR9** | | Toll-like receptor 9 |  |
| *TNF** | | Tumor necrosis factor |  |
| *TNFRSF1A* | | Tumor necrosis factor receptor superfamily, member 1A |  |
| *TOLLIP* | | Toll interacting protein |  |
| *TRAF6** | | TNF receptor-associated factor 6 |  |
| *XIAP* | | X-linked inhibitor of apoptosis |  |
| *ZBP1* | | Z-DNA binding protein 1 |  |
|  | | ***Innate and Adaptive Immune Responses*** |  |
| **Gene name** | | **Description** |  |
| *APCS*^#^* | | Amyloid P component, serum |  |
| *C3* | Complement component 3 | | |
| *CASP1** | Caspase 1, apoptosis-related cysteine peptidase (interleukin 1, beta, convertase) | | |
| *CCL2* | Chemokine (C-C motif) ligand 2 | | |
| *CCL5** | Chemokine (C-C motif) ligand 5 | | |
| *CCR4* | Chemokine (C-C motif) receptor 4 | | |
| *CCR5* | Chemokine (C-C motif) receptor 5 | | |
| *CCR6* | Chemokine (C-C motif) receptor 6 | | |
| *CCR8^#^* | Chemokine (C-C motif) receptor 8 | | |
| *CD14** | CD14 molecule | | |
| *CD4* | CD4 molecule | | |
| *CD40* | CD40 molecule, TNF receptor superfamily member 5 | | |
| *CD40LG* | CD40 ligand | | |
| *CD80* | CD80 molecule | | |
| *CD86* | CD86 molecule | | |
| *CD8A* | CD8a molecule | | |
| *CRP*^#^* | C-reactive protein, pentraxin-related | | |
| *CSF2* | Colony stimulating factor 2 (granulocyte-macrophage) | | |
| *CXCL10* | Chemokine (C-X-C motif) ligand 10 | | |
| *CXCR3* | Chemokine (C-X-C motif) receptor 3 | | |
| *DDX58* | DEAD (Asp-Glu-Ala-Asp) box polypeptide 58 | | |
| *FASLG* | Fas ligand (TNF superfamily, member 6) | | |
| *FOXP3* | Forkhead box P3 | | |
| *GATA3* | GATA binding protein 3 | | |
| *HLA-A* | Major histocompatibility complex, class I, A | | |
| *HLA-E* | Major histocompatibility complex, class I, E | | |
| *ICAM1* | Intercellular adhesion molecule 1 | | |
| *IFNA1*^#^* | Interferon, alpha 1 | | |
| *IFNAR1* | Interferon (alpha, beta and omega) receptor 1 | | |
| *IFNB1*^#^* | Interferon, beta 1, fibroblast | | |
| *IFNG* | Interferon, gamma | | |
| *IFNGR1* | Interferon gamma receptor 1 | | |
| *IL10* | Interleukin 10 | | |
| *IL13* | Interleukin 13 | | |
| *IL17A^#^* | Interleukin 17A | | |
| *IL18** | Interleukin 18 (interferon-gamma-inducing factor) | | |
| *IL1A* | Interleukin 1, alpha | | |
| *IL1B** | Interleukin 1, beta | | |
| *IL1R1* | Interleukin 1 receptor, type I | | |
| *IL2* | Interleukin 2 | | |
| *IL23A* | Interleukin 23, alpha subunit p19 | | |
| *IL4^#^* | Interleukin 4 | | |
| *IL5* | Interleukin 5 (colony-stimulating factor, eosinophil) | | |
| *IL6** | Interleukin 6 (interferon, beta 2) | | |
| *CXCL8** | Interleukin 8 | | |
| *IRAK1** | Interleukin-1 receptor-associated kinase 1 | | |
| *IRF3* | Interferon regulatory factor 3 | | |
| *IRF7** | Interferon regulatory factor 7 | | |
| *ITGAM* | Integrin, alpha M (complement component 3 receptor 3 subunit) | | |
| *JAK2* | Janus kinase 2 | | |
| *LY96** | Lymphocyte antigen 96 | | |
| *LYZ** | Lysozyme | | |
| *MAPK1** | Mitogen-activated protein kinase 1 | | |
| *MAPK8** | Mitogen-activated protein kinase 8 | | |
| *MBL2^#^* | Mannose-binding lectin (protein C) 2, soluble | | |
| *MPO*^#^* | Myeloperoxidase | | |
| *MX1* | Myxovirus (influenza virus) resistance 1, interferon-inducible protein p78 (mouse) | | |
| *MYD88** | Myeloid differentiation primary response gene (88) | | |
| *NFKB1** | Nuclear factor of kappa light polypeptide gene enhancer in B-cells 1 | | |
| *NFKBIA** | Nuclear factor of kappa light polypeptide gene enhancer in B-cells inhibitor, alpha | | |
| *NLRP3** | NLR family, pyrin domain containing 3 | | |
| *NOD1** | Nucleotide-binding oligomerization domain containing 1 | | |
| *NOD2** | Nucleotide-binding oligomerization domain containing 2 | | |
| *RAG1* | Recombination activating gene 1 | | |
| *RORC* | RAR-related orphan receptor C | | |
| *SLC11A1** | Solute carrier family 11 (proton-coupled divalent metal ion transporters), member 1 | | |
| *STAT1* | Signal transducer and activator of transcription 1, 91kDa | | |
| *STAT3* | Signal transducer and activator of transcription 3 (acute-phase response factor) | | |
| *STAT4* | Signal transducer and activator of transcription 4 | | |
| *STAT6* | Signal transducer and activator of transcription 6, interleukin-4 induced | | |
| *TBX21* | T-box 21 | | |
| *TICAM1** | Toll-like receptor adaptor molecule 1 | | |
| *TLR1** | Toll-like receptor 1 | | |
| *TLR2** | Toll-like receptor 2 | | |
| *TLR3* | Toll-like receptor 3 | | |
| *TLR4** | Toll-like receptor 4 | | |
| *TLR5** | Toll-like receptor 5 | | |
| *TLR6** | Toll-like receptor 6 | | |
| *TLR7* | Toll-like receptor 7 | | |
| *TLR8* | Toll-like receptor 8 | | |
| *TLR9** | Toll-like receptor 9 | | |
| *TNF** | Tumor necrosis factor | | |
| *TRAF6** | TNF receptor-associated factor 6 | | |
| *TYK2* | Tyrosine kinase 2 | | |
| **Gene name** | | ***Housekeeping genes*** |  |
| *ACTB* | | Actin, beta |  |
| *B2M* | | Beta-2-microglobulin |  |
| *GAPDH* | | Glyceraldehyde-3-phosphate dehydrogenase |  |
| *HPRT1* | | Hypoxanthine phosphoribosyltransferase 1 |  |
| *RPLP0* | | Ribosomal protein, large, P0 |  |

^#^Gene not detected in the analysis.

*Gene present in both arrays.
